# Leprosy-Related Stigma and Social Representations Among Persons Affected by Leprosy and Primary Healthcare Professionals: A Structural Analysis in the Brazilian Amazon

**DOI:** 10.1007/s10900-026-01618-2

**Published:** 2026-07-25

**Authors:** Francisco Albino de Araújo, Vinicius Silva dos Santos, Woska Pires da Costa, Eloiza Toledo Bauduina, Italla Maria Pinheiro Bezerra, Luiz Carlos de Abreu

**Affiliations:** 1 Post-Graduate Program in Health Sciences, Faculdade de Medicina do ABC (FMABC), Príncipe de Gales Avenue, No. 821, Vila Príncipe de Gales, SP 09060-650 Santo André, Brazil; 2 Instituto de Ciências Exatas e Naturais (ICEN), Universidade Federal do Pará (UFPA), Augusto Corrêa Street, No. 1, Guamá University Campus, PA 66075-110 Belém, Brazil; 3 Research Department, Instituto Federal Goiano - Campus Morrinhos, BR-153 Highway, Km 633, Rural Area, PO Box 92, Morrinhos, 75658-899 GO Brazil; 4 Medical School, Universidade de São Paulo (USP), Dr. Arnaldo Avenue, No. 455, Cerqueira César Neighborhood, Central Institute of Clinical Hospital Building, Block II, 2nd Floor, PO Box 8,091, São Paulo, 01246-903 SP Brazil; 5 Post-Graduate Program in Public Policy and Local Development, Escola Superior de Ciências da Santa Casa de Misericórdia de Vitória (EMESCAM), Nossa Senhora da Penha Avenue, Santa Luíza Neighborhood, Vitória, 29045-402 ES Brazil; 6 Department of Nutrition, Universidade Federal do Espírito Santo (UFES), Marechal Campos Avenue, No. 1468, Maruípe, Vitória, 29040-090 ES Brazil; 7 Center for Health and Sports Sciences (CCSD), Universidade Federal do Acre (UFAC), BR-364 Highway, Km 04, Industrial District Neighborhood, Rio Branco, 69915-900 AC Brazil; 8 Department of Rehabilitation Sciences, Florida Gulf Coast University (FGCU), FGCU Boulevard South, No. 10501, FL 33965-6565 Fort Myers, United States; 9 University of Limerick (UL), Castletroy, Co., Limerick, V94 T9PX Ireland

**Keywords:** Leprosy, Neglected tropical disease, Stigma, Social representations, Primary healthcare, Diagnostic delay

## Abstract

Leprosy remains a persistent public health challenge not only because transmission continues in endemic settings, but also because stigma remains embedded in social relations, institutional practices, and historical forms of exclusion. This study analyzes the social representations of leprosy among persons affected by leprosy and primary healthcare professionals in a historically endemic area of the Brazilian Amazon, with particular attention to how these representations shape disease recognition, stigma, and care. An exploratory mixed-methods design combined structured questionnaires with a Free Word Association Test to investigate shared meanings of leprosy among 34 persons affected by leprosy (age available for 23 participants; range, 20–85 years) and 19 primary healthcare professionals. Findings show that leprosy is socially understood less through biomedical curability than through stigma, visible bodily damage, and exclusionary meanings, reflected in candidate central elements such as “prejudice”, “deformity”, and “pain”. Although most affected participants recognized that leprosy is curable, substantial knowledge gaps were identified, particularly regarding transmission pathways. Similar limitations were observed among healthcare professionals, indicating weaknesses in diagnostic preparedness within primary healthcare. These results suggest that stigma in leprosy cannot be reduced to insufficient knowledge alone, but should instead be understood as a biosocial phenomenon produced through the interaction of social meanings, embodied experience, and institutional conditions. These dynamics may contribute to delayed diagnosis and reinforce the continued neglect of the disease, requiring integrated strategies that go beyond biomedical control, strengthen primary healthcare, and transform the social and institutional conditions that sustain stigma, diagnostic delay, and exclusion.

**Supplementary Information:**

The online version contains supplementary material available at https://doi.org/10.1007/s10900-026-01618-2.

## Introduction

Despite the availability of effective treatment, leprosy remains a significant public health problem worldwide. More than a biomedical condition, leprosy is a socially embedded disease whose meanings, trajectories, and consequences are shaped by stigma, exclusion, and institutional responses that co-produce how illness is recognized and managed. Historically known as Hansen’s disease, leprosy is a chronic infectious condition caused primarily by *Mycobacterium leprae* (*M. leprae*) [1–3] and, more recently, also associated with *Mycobacterium lepromatosis* (*M. lepromatosis*), both of which affect the peripheral nerves and skin [2–4]. Although curable, delayed diagnosis may result in irreversible disability, particularly in socially vulnerable populations [5–8].

Leprosy is classified among Neglected Tropical Diseases (NTDs), which disproportionately affect populations exposed to poverty and restricted access to healthcare, reflecting the inseparability of biological vulnerability and social neglect [2, 9, 10]. Its persistence reflects not only biological factors but also social conditions that sustain transmission, delay care-seeking, and reproduce inequalities in health outcomes. These dynamics reflect broader processes through which structural inequalities and institutional arrangements shape patterns of disease recognition and healthcare access. Global estimates indicate a reduction in incidence between 1990 and 2019, yet the disease remains concentrated in endemic regions such as Africa and Southeast Asia, with underreporting masking its true burden [1, 7, 10, 11]. Brazil occupies a particularly important place in this epidemiological landscape, both because of its sustained burden of new cases and because it offers a critical setting for examining how endemicity, inequality, and institutional history intersect. The country ranks among those with the highest numbers of new cases worldwide and accounts for the overwhelming majority of reported cases in the Americas, particularly in the North, Northeast, and Central-West regions [7, 12–14]. In 2023, 182,815 new cases of leprosy were reported globally, of which Brazil accounted for 22,773 cases, corresponding to 92% (22,773 cases) of all cases reported in the Americas [13].

In Brazil, diagnosis is primarily clinical and remains challenging in primary healthcare, especially when early or atypical presentations are encountered in settings marked by limited training, diagnostic uncertainty, and uneven service capacity. This is particularly evident in atypical neural or dermatoneurological presentations, which may lead to diagnostic uncertainty, especially with limited training or misdiagnosis as other neuropathies or dermatoses [5, 8]. Delayed diagnosis remains associated with high rates of grade II disabilities (G2D), reinforcing the importance of early detection [6, 15, 16]. However, the persistence of leprosy cannot be explained solely by diagnostic or therapeutic limitations. Stigma remains a central dimension of the disease, historically rooted in isolation, social exclusion, and moral judgment, and it continues to shape symptom interpretation, healthcare encounters, and institutional responses well beyond the biomedical domain. The legacy of segregation in leprosaria, combined with persistent misinformation and fear, has produced enduring negative meanings shared by individuals, professionals, and communities. These meanings actively shape symptom recognition, care-seeking, and interactions within healthcare systems [17–19].

The World Health Organization (WHO) has emphasized that effective leprosy control requires more than the availability of treatment; it also depends on stigma reduction, community education, and socially responsive care [7, 17, 20]. This underscores the need to examine not only knowledge but also how leprosy is interpreted and experienced in everyday life, particularly in historically endemic contexts where collective memories of segregation persist.

In this regard, Social Representations Theory provides a particularly relevant framework for examining how leprosy is collectively interpreted, stabilized, and communicated in everyday life. Proposed by Moscovici [21], this theory conceptualizes representations as socially constructed systems of knowledge that shape behavior and communication. Within the structural approach, social representations (SR) are organized around a stable central core and a flexible peripheral system [22], allowing the analysis of how meanings are maintained and adapted in context. In leprosy, these representations may influence diagnostic trajectories, treatment adherence, and care practices [17, 18]. This perspective is particularly relevant for examining how social meanings and institutional capacities are intertwined in shaping health outcomes.

Although previous studies have examined leprosy-related stigma, knowledge, and community attitudes, fewer have explored how SR are structurally organized across both persons affected by leprosy and frontline primary healthcare professionals, particularly in historically endemic settings where institutional memory remains socially active. Addressing this gap is especially important in regions where leprosy remains socially visible, institutionally remembered, and epidemiologically relevant.

Against this background, this study analyzes the SR of leprosy among persons affected by leprosy and primary healthcare professionals in the Brazilian Amazon, focusing on how socially constructed meanings shape disease recognition, care-seeking, and professional practice. The study contributes to international debates by conceptualizing leprosy as a biosocial phenomenon in which stigma, embodied visibility, and diagnostic capacity are co-produced within historically structured health systems. The findings also highlight how primary healthcare fragilities and institutional memory influence disease recognition and care in peripheral contexts, reinforcing the need to address stigma, inequality, and the social meanings of illness in leprosy control.

## Method

### Study Design

This study adopted an exploratory mixed-methods design with integration at the interpretive level, combining structured questionnaire data, evocation-based measures, and narrative excerpts to examine both the shared semantic structure and the socially situated meanings of leprosy. The Free Word Association Test (FWAT), widely employed in studies of SR [23], was used as a projective technique to access spontaneous and socially shared associations related to leprosy [24, 25]. Grounded in the central core theory [26], prototypical analysis of evoked terms enables the identification of candidate central and peripheral elements based on the frequency and order of evocation.

Prototypical analysis combined frequency and mean order of evocation to identify candidate central and peripheral elements within the representational structure. The design integrated quantitative indicators of representational salience, including frequency and mean order of evocation, with qualitative material reflecting lived experience and attributed meaning, thereby enabling a more analytically layered understanding of leprosy as both a symbolic and embodied condition. Within the SR framework, such triangulation enables the examination of shared semantic structures alongside socially situated meanings [21, 27, 28], enhancing the analysis of complex health-related phenomena through complementary forms of evidence [29, 30].

This approach is particularly relevant for leprosy, a condition historically shaped by stigma and socially embedded narratives influencing health-seeking behaviors and professional practices. By capturing spontaneous associations, the FWAT links structural indicators with the symbolic and experiential dimensions of the disease.

### Ethical Approval

The study was conducted in accordance with the ethical principles of the Declaration of Helsinki [31] and Brazilian national research regulations. Ethical approval was obtained from the Research Ethics Committee of the University Center of the *Faculdade de Medicina do ABC* (*FMABC*), Protocol *CAAE* No. 48612521.9.0000.0082, Opinion No. 5.111.332, of November 18, 2021. All the recommendations contained in the Brazilian Resolution *CNS* No. 466, of December 12, 2012, of the National Health Council [32], were strictly followed throughout all stages of the research.

### Study Setting

The study was conducted in the community surrounding the former leprosarium of Cruzeiro do Sul, in the state of Acre, Brazilian Amazon, a setting of particular analytical relevance due to its historical association with compulsory isolation and leprosy care. The site currently houses the *Hospital de Dermatologia Sanitária*, a historical reference center for the treatment and follow-up of individuals affected by leprosy. The choice of this setting was analytically relevant given its long-standing institutional and social association with the management of the disease. Historically, leprosaria functioned not only as sites of medical care but also as spaces of compulsory segregation, contributing to the formation of collective memories linked to stigma, exclusion, and restricted access to healthcare.

Within the theoretical framework of SR, meanings attributed to social objects are shaped by historically situated experiences and broader sociocultural contexts [27]. In this perspective, places historically associated with disease management may play a significant role in shaping the symbolic and experiential dimensions through which individuals interpret illness. The legacy of institutional isolation policies implemented throughout the twentieth century may therefore continue to influence how leprosy is socially understood and remembered in communities historically connected to leprosaria.

The epidemiological context further reinforces this relevance. Brazil accounted for approximately 92% of new leprosy cases reported in the Americas in 2023 [13], with higher detection rates concentrated in the North, Northeast, and Central-West regions of the country [9, 33]. A leprosarium was established in Cruzeiro do Sul in 1927 during a period of high endemicity [34]. This historical legacy contributes to a collective memory of segregation that may still shape contemporary social representations of leprosy within the region.

### Participants and Sampling Strategy

Participants were recruited from two groups directly related to the phenomenon under study: (1) persons affected by leprosy and (2) primary healthcare professionals working in municipal Basic Health Units (BHUs). Sampling procedures differed between groups. Persons affected by leprosy were recruited purposively because direct lived experience was central to the research question. Primary healthcare professionals were recruited from selected rural and urban BHUs to capture variation across professional roles and service contexts. These approaches prioritized analytically relevant perspectives rather than statistical representativeness [35, 36].

Eligibility criteria were defined separately for each group. Persons affected by leprosy were eligible if they were aged 18 years or older, had a confirmed diagnosis of leprosy, resided in the municipality, and voluntarily consented to participate. Primary healthcare professionals were eligible if they worked in one of the selected BHUs in a role potentially related to leprosy prevention, diagnosis, treatment, surveillance, or management; prior direct experience with leprosy was not required. Persons residing outside the municipality or unable to complete the study procedures were not enrolled.

#### Persons Affected by Leprosy

Municipal surveillance records documented 642 leprosy diagnoses in Cruzeiro do Sul between 2001 and 2021 (424 men and 218 women). These records were used solely to characterize the recent epidemiological context and did not constitute the sampling frame. The analytical sample comprised 34 persons affected by leprosy, including individuals diagnosed before 2001, who were purposively recruited among those who attended the community healthcare initiative. Epidemiological data indicating that 32% of cases among men and 43% among women were detected through contact examinations were used only to contextualize sex-specific patterns of case detection [15]. No probability-based sample size calculation was applied to this group.

#### Healthcare Professionals

Healthcare professionals were recruited from selected Basic Health Units serving rural and urban areas. Nineteen eligible professionals who were available and consented to participate were included. The service-based, non-probability sample was intended to capture variation across professional roles and service contexts rather than to support population-level estimates.

### Data Collection Procedure

Data collection with persons affected by leprosy took place during a free collective healthcare service held on April 29, 2022, organized in collaboration with medical students from a local college. After written informed consent was obtained, participants completed an individual structured interview based on pre-established guiding questions, followed by the FWAT using the inducing term “leprosy” [23]. Participants were asked to evoke words or expressions that immediately came to mind in relation to the inducing term. The order of evocation was recorded exactly as produced, and participants were subsequently asked to rank the evoked terms by perceived importance.

Interviews and FWAT procedures were conducted individually in a quiet, reserved area within the healthcare facility to ensure privacy and minimize external influences on participants’ responses. Each interview lasted approximately 10 min and included questions on sociodemographic characteristics, illness experiences, and perceptions of leprosy. The instrument administered to persons affected by leprosy is provided in Online Resource 1, and the instrument administered to healthcare professionals is provided in Online Resource 2. Following the interview and FWAT procedure, affected participants underwent a physical examination conducted by a physiotherapist and a subsequent medical evaluation performed by a physician participating in the healthcare initiative. Healthcare professionals completed the same FWAT and structured interviews individually in their workplace. Their structured interviews focused on professional roles, experiences, and practices related to the prevention, diagnosis, and management of leprosy in primary healthcare services. Procedures were standardized across both participant groups to enhance comparability in the elicitation of SR.

All interviewers received prior training to standardize instrument administration and ensure consistency in instructions provided to participants. The lead researcher supervised fieldwork.

### Free Word Association Test (FWAT)

The FWAT, known in Brazil as *Teste de Associação Livre de Palavras* (*TALP*), is widely used in SR studies to access semantic and cognitive contents through spontaneous evocations [28, 37]. In this procedure, participants are invited to report the first words or expressions that come to mind in response to a given inducing term, allowing researchers to identify salient representational elements and networks of meaning associated with the object under investigation [25, 38, 39]. Participants were asked to evoke five words related to “leprosy” and rank them by importance, following established procedures [40, 41]. This enabled the identification of salient representational elements.

### Prototypical Analysis of Social Representations

Data analysis followed the structural approach to SR proposed by Abric [42], which distinguishes between the central core and the peripheral elements. The central core comprises the most stable and socially shared elements of a representation, reflecting the collective meanings constructed within a given social group; in contrast, the peripheral system incorporates more flexible and context-sensitive elements that allow individual interpretations and situational adaptations while preserving the stability of the representational structure [42]. This dynamic relationship between central and peripheral elements allows representations to remain socially shared while also accommodating contextual variation and individual experience [43, 44].

**Fig. 1:**
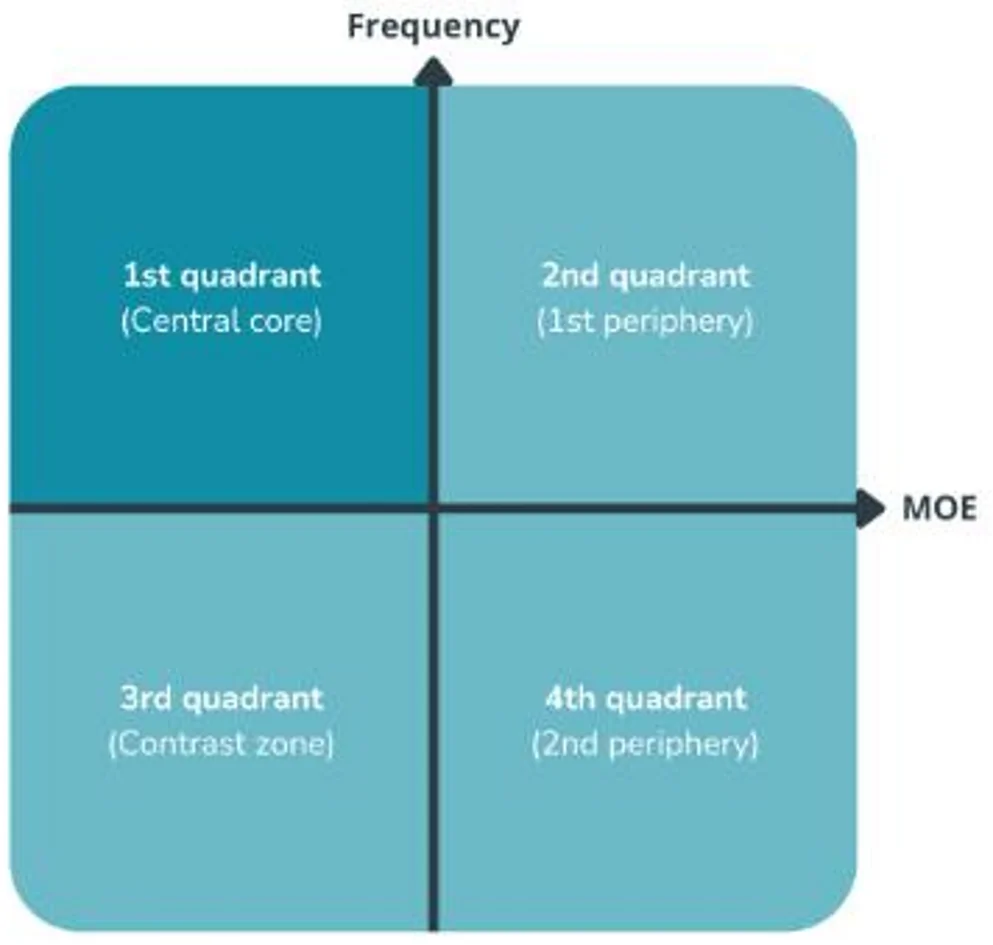
Quadrants used in the prototypical analysis of Free Word Association Test responses according to frequency and mean order of evocation

The structural characterization of the SR of leprosy was conducted using the FWAT. Participants were asked to evoke five words associated with the inducing term “leprosy”, thereby exploring the semantic relationships between evoked elements and the social object under investigation [45]. The evoked terms were analyzed by frequency of occurrence and mean order of evocation (MOE), thereby enabling the identification of salient representational elements. Following the prototypical analysis proposed by Vergès [23], evoked terms were organized into a four-quadrant matrix combining frequency and MOE (Fig. 1). This analytical procedure enables the identification of candidate structural positions within the representational field.

The quadrants are organized as follows [45]:

- Upper-left: candidate central core (high frequency, low MOE), comprising early and widely shared terms that structure and stabilize the representation in relation to the peripheral system [46].
- Upper-right: 1 st periphery (high frequency, high MOE), including frequently mentioned but later-evoked elements.
- Lower-left: contrast zone (low frequency, low MOE), comprising less frequent but salient terms for specific participants.
- Lower-right: 2nd periphery (low frequency, high MOE), reflecting more individualized or context-specific elements [22].

To further examine the internal organization of the representation, FWAT responses were analyzed using combined Rank–Frequency and Importance–Frequency procedures [40], which integrate term frequency, evocation order, and perceived importance. This approach enables the identification of elements that are both cognitively salient and symbolically relevant within the representational structure.

All analyses were conducted using the *TELP* package (*Técnica de Evocação Livre de Palavras* – in English: Free Word Evocation Technique), in the R environment [45, 47], which computes frequency and mean order of evocation and allocates terms into the four quadrants according to standard prototypical analysis criteria.

Before prototypical analysis, spelling variants, singular and plural forms, and semantically equivalent expressions were harmonized using a documented coding dictionary, while conceptually distinct expressions were retained as separate categories. One healthcare-professional response (“cinco” [“five”]) could not be interpreted as a meaningful leprosy-related evocation and was excluded from the prototypical analysis. The complete coding dictionary is provided in the accompanying dataset.

Descriptive statistics were calculated using available-case denominators. Responses concerning clinical forms and recommended multidrug treatment regimens were analyzed only among physicians (*n* = 7), because these items were considered within their professional scope.

## Results

The sample comprised 34 persons affected by leprosy. Age data were available for 23 participants, whose ages ranged from 20 to 85 years (mean 63.43, *SD* 16.96). Most participants were male (64.7%) and self-identified as Black or Mixed (pardo) (64.7%). Overall, 55.9% reported no schooling, literacy-only education, or incomplete primary schooling. Additionally, 52.9% reported living with a spouse or partner, and 64.7% did not work outside the home; 14 participants (41.2%) explicitly reported being retired (Table 1).

**Table 1:** Sociodemographic profile of persons affected by leprosy

Variable	*N*	%	Variable	*N*	%
**Sex**			**Works outside the home?**		
Female	12	35.3	Yes	10	29.4
Male	22	64.7	No	22	64.7
			Not informed	2	5.9
**Ethnicity/skin color** ^a^			**How did you hear about the disease?**		
Yellow	2	5.9	Someone in the family	11	32.4
White	9	26.5	At the health center	8	23.5
Black or Mixed	22	64.7	At work	2	5.9
Not informed	1	2.9	Never heard of it	2	5.9
			Other	11	32.4
**Education level**			**Time to seek help**		
No schooling	8	23.5	Immediate	5	14.7
Elementary school (incomplete)	11	32.4	≤ 6 months	8	23.5
Elementary school (complete)	9	26.5	1 to 2 years	5	14.7
High school (incomplete)	1	2.9	2 to 5 years	5	14.7
High school (complete)	3	8.8	> 5 years	7	20.6
Higher education (incomplete)	2	5.9	Don’t know how to answer	4	11.8
**Who do you live with?**			**Evaluation of healthcare received**		
Spouse/Partner	18	52.9	Great/Excellent	4	11.8
Children	8	23.5	Good	21	61.8
Other	2	5.9	Regular	5	14.7
Relatives	1	2.9	Poor/Very Poor	3	8.8
Alone	5	14.7	Not informed	1	2.9
**Total**	**34**	**100.0**	**Total**	**34**	**100.0**

Note: ^a^ Ethnicity/skin color was self-identified according to Brazilian census classifications (*Instituto Brasileiro de Geografia e Estatística* – *IBGE*) and reported using internationally comparable terminology, adapted to recognized classifications: White (Caucasian origin), Black (African descent), Yellow (East Asian ancestry), and Mixed ethnicity to the Brazilian “*Pardo*” category, encompassing individuals with diverse ancestral backgrounds [48]

Time since diagnosis was interpretable for 31 participants and varied widely (mean 40.56 years, *SD* 25.28; range, diagnosis on the day of data collection to 73 years). Approximately one-third of participants reported learning about the disease through family members. Time to care-seeking varied, with 38.2% seeking care within 6 months of symptom onset and 20.6% after more than 5 years. Most participants (73.5%) rated the care provided by healthcare professionals as good, great, or excellent.

Among the 19 healthcare professionals, participants included physicians, nurses, nursing technicians, community health workers, primary healthcare managers, and one physiotherapist (Table 2). Age data were available for 18 participants; ages ranged from 24 to 52 years (mean 40.33, *SD* 8.28). Length of service ranged from 1 to 2 months to 28 years (mean 10.86 years, *SD* 9.30). Two participants had not received formal training in leprosy, although all identified at least one sign or symptom. Among 18 respondents, two (11.1%) reported prior experience diagnosing or reporting a suspected leprosy case; one response was missing. Nearly half (47.4%) reported no involvement in leprosy-related activities. Among the seven physicians, five (71.4%) identified the expected treatment regimens, two (28.6%) selected other regimens, and three (42.9%) reported not knowing the clinical forms of leprosy.

**Table 2:** Characterization of healthcare professionals in relation to leprosy-related clinical variables

Variable	*N*	%	Variable ^b^	*N*	%
**Healthcare professionals**			**Recommended treatment for paucibacillary form**		
Community health worker	2	10.5	Rifampicin alone	–	–
Nurse	4	21.1	Rifampicin and Clofazimine	–	–
Physician	7	36.8	Rifampicin and Dapsone	2	28.6
Nursing technician	3	15.8	Rifampicin, Clofazimine, and Dapsone	5	71.4
Other ^a^	3	15.8			
**Actions taken**			**Recommended treatment for multibacillary form**		
Provides prevention guidance	4	21.1	Rifampicin only	–	–
Identifies suspected cases	2	10.5	Clofazimine only	–	–
Provides educational activities	3	15.8	Dapsone only	2	28.6
Conducts home visits	1	5.3	Rifampicin, Clofazimine, and Dapsone	5	71.4
None	9	47.4	**Knowledge of clinical forms**		
			Yes	4	57.1
			No	3	42.9
**Total**	19	100.0	**Total**	7	100.0

Note: ^a^ Primary Health Care (PHC) unit manager/physiotherapist. ^b^ Analyses of knowledge of clinical forms and recommended treatment regimens were restricted to physicians (*n* = 7), because these items were considered within their professional scope

Most affected participants (85.3%) and healthcare professionals (94.7%) recognized that leprosy is curable. However, 82.4% of affected participants did not know how leprosy is transmitted. Among the six who reported knowing, two clearly referred to close contact or respiratory transmission, one referred more broadly to an oral route, and the remaining responses cited heredity, shared surfaces, or hygiene. Among healthcare professionals, three (15.8%) reported not knowing how leprosy is transmitted; responses from the remaining 16 included close contact, respiratory droplets, prolonged contact, and nonspecific or incomplete descriptions of transmission.

Regarding follow-up of household and close contacts, 12 affected participants (35.3%) and eight healthcare professionals (42.1%) reported that such care was never provided (Table 3).

**Table 3:** Knowledge of transmission and cure and reported contact follow-up among affected participants and healthcare professionals

Variable	Affected participants		Healthcare professionals	
	*N*	%	*N*	%
**Reported knowledge of leprosy transmission**				
Yes	6	17.6	16	84.2
Does not know	28	82.4	3	15.8
**Is leprosy curable?**				
Yes	29	85.3	18	94.7
No	5	14.7	1	5.3
**Was follow-up care provided to household and close contacts?**				
Always	9	26.5	5	26.3
Often	2	5.9	6	31.6
Occasionally	2	5.9	–	–
Rarely	4	11.8	–	–
Never	12	35.3	8	42.1
Not informed	5	14.7	–	–
**Total**	**34**	**100.0**	**19**	**100.0**

Affected participants evoked 22 distinct terms in response to the stimulus “leprosy”; “prejudice” and “isolation” were the most frequent (Table 4). For this group, the quadrant thresholds were frequency 6 and MOE 3.00.

**Table 4:** Quadrants of terms evoked by affected participants in response to “leprosy” in Cruzeiro do Sul, Acre, in 2022

1st quadrant – Candidate central core (Frequency ≥ 6 and MOE < 3)			2nd quadrant – 1st periphery (Frequency ≥ 6 and MOE ≥ 3)		
Term evoked	Frequency	MOE	Term evoked	Frequency	MOE
Prejudice	23	2.57	Isolation	21	3.19
Deformity	15	2.80	Lesion	12	3.00
Pain	14	2.79	Treatment	10	3.90
Cure	8	2.12	Sadness	10	3.30
Disease	8	2.25	Stigma	7	3.43
			Deprivation	6	3.83

3rd quadrant – Contrast zone (Frequency < 6 and MOE < 3)			4th quadrant – 2nd periphery (Frequency < 6 and MOE ≥ 3)		
Term evoked	Frequency	MOE	Term evoked	Frequency	MOE
Leprosy	5	2.40	Family	5	4.20
Incurable	4	1.50	Fear	5	4.00
Transmission	4	2.50	Lack of knowledge	3	3.67
Acceptance	1	1.00	Concern	3	3.00
			Spot	2	3.50
			Non-infectious	2	3.00
			Susceptible	1	5.00

Note: MOE stands for Mean Order of Evocation

Healthcare professionals produced 95 evocations. One uninterpretable response was excluded, leaving 94 valid evocations grouped into 18 harmonized terms. The words “lesion” and “prejudice” were the most frequent terms (Table 5). The cut-off points for frequency and MOE were 4 and 3.34, respectively.

**Table 5:** Quadrants of terms evoked by healthcare professionals in response to “leprosy” in Cruzeiro do Sul, Acre, in 2022

1st quadrant – Candidate central core (Frequency ≥ 4 and MOE < 3.34)			2nd quadrant – 1st periphery (Frequency ≥ 4 and MOE ≥ 3.34)		
Term evoked	Frequency	MOE	Term evoked	Frequency	MOE
Lesion	16	2.56	Transmission	7	3.43
Prejudice	14	2.93	Isolation	5	3.80
Spot	9	1.56	Fear	4	3.50
Deformity	8	3.12	Pain	4	4.00
Treatment	8	3.25			
Disease	5	2.60			

3rd quadrant – Contrast zone (Frequency < 4 and MOE < 3.34)			4th quadrant – 2nd periphery (Frequency < 4 and MOE ≥ 3.34)		
Term evoked	Frequency	MOE	Term evoked	Frequency	MOE
Stigma	3	2.00	Deprivation	3	3.67
Leprosy	1	2.00	Sadness	2	3.50
Incurable	1	3.00	Cure	2	5.00
			Lack of knowledge	1	4.00
			Neglect	1	5.00

Note: MOE stands for Mean Order of Evocation

FWAT findings identified “prejudice”, “deformity”, “pain”, “cure”, and “disease” as candidate central elements among affected participants. Among healthcare professionals, candidate central elements included “lesion”, “prejudice”, “spot”, “deformity”, “treatment”, and “disease”. The presence of “prejudice” among the candidate central elements in both groups underscores the salience of stigma within the representational field.

One affected participant described concealment and rejection associated with visible impairments:

- “*Sempre escondi as mãos*,* lembro que uma vez pediram ajuda*,* mas quando viram minhas mãos*,* recusaram ajuda; não me cumprimentam na igreja; dizem lá vem o leproso.*” —translated: “*I have always hidden my hands*,* I remember once they asked for help*,* but when they saw my hands*,* they refused help; they don’t greet me in church; they say here comes the leper.*”

Another affected participant recalled discriminatory treatment in everyday commercial and social interactions:

- “*Ninguém queria pegar meu dinheiro; nós temos ordem para não servir pessoas assim; a pensão mudou*,* mas as mãos ficaram aleijadas; nunca mais comi fora*,* deixei o dinheiro da xícara e do tamborete.*”—translated: “*Nobody wanted to take my money; we were ordered not to serve people like that; the pension changed*,* but my hands were crippled; I never ate out again*,* I left the money in the cup and on the stool.*”

A further account described childhood institutionalization and severe, permanent physical impairments that restricted mobility, independence, and social participation. To reduce the risk of indirect re-identification, this experience is presented as a paraphrase rather than as a verbatim quotation.

- “[…] *a princípio com 12 anos*,* só queria brincar*,* depois fiquei internada em isolamento do mundo desde então e hoje*,* só quero ter minha liberdade*,* pois vivo presa a uma cadeira de rodas*,* sem pernas e com minhas mãos mutiladas.*”—translated: “[…] *at first*,* when I was 12*,* I just wanted to play*,* then I was interned in isolation from the world ever since and today*,* I just want to have my freedom*,* because I’m stuck in a wheelchair*,* with no legs and my hands mutilated.*”

Among affected participants, the first periphery comprised “isolation”, “lesion”, “treatment”, “sadness”, “stigma”, and “deprivation” (Table 4). Among healthcare professionals, it comprised “transmission”, “isolation”, “fear”, and “pain” (Table 5). In the second periphery, affected participants evoked “family”, “fear”, “lack of knowledge”, “concern”, “spot”, “non-infectious”, and “susceptible”. The occurrence of “lack of knowledge” in this peripheral zone complements the questionnaire findings on transmission.

Among healthcare professionals, the second periphery included “deprivation”, “sadness”, “cure”, and “lack of knowledge”. Because prototypical analysis classifies terms by frequency and mean order of evocation, quadrant placement should not be interpreted as evidence of statistical proximity or semantic clustering.

## Discussion

### Leprosy as a Socially Mediated Disease

This study shows that leprosy continues to be represented through stigma, bodily damage, and exclusionary meanings rather than primarily through biomedical understandings of cure. Among affected participants, candidate central elements included “prejudice”, “deformity”, “pain”, “cure”, and “disease”; among healthcare professionals, they included “lesion”, “prejudice”, “spot”, “deformity”, “treatment”, and “disease”. The prominence of “prejudice” in both groups indicates that stigma remains an important organizing principle in collective understandings of leprosy. “Isolation”, although located in the first periphery, was frequent in both groups and connects shared representations with lived experiences of exclusion.

These findings are consistent with previous research showing that leprosy remains strongly associated with stigma, misinformation, and social exclusion across diverse contexts [17, 19, 49, 50]. Individuals frequently report social rejection, concealment of symptoms, and delayed care seeking due to fear of discrimination [19, 51, 52], patterns also observed in other neglected tropical diseases [53, 54].

From the perspective of Social Representations Theory, the prominence of stigma-related elements identifies them as candidates for the central core of the representational structure. The central core represents the most stable and consensual interpretations shared by a social group and plays a key role in organizing how a phenomenon is interpreted in everyday life [22, 26, 28]. However, prototypical analysis alone does not confirm centrality; complementary procedures would be required to establish that prejudice, deformity, and pain occupy this structural position [21].

The frequency of these terms reinforces this interpretation. Among affected participants, “prejudice” (23), “isolation” (21), “deformity” (15), and “pain” (14) were most cited. The presence of “prejudice” among professionals (14 mentions) suggests that stigma also circulates within healthcare settings [50, 53]. These findings indicate that interventions based solely on biomedical information may be insufficient and may require strategies that address the symbolic dimensions that sustain stigma.

### Embodied Stigma and the Social Visibility of Disease

The prominence of terms such as “deformity”, “lesion”, “pain”, and “spot” highlights the role of bodily manifestations in shaping social perceptions of leprosy. The disease was represented as a visible and embodied condition in which the body becomes a primary site of recognition, judgment, and stigma. This interpretation aligns with Goffman’s concept of the “spoiled identity”, according to which visible bodily differences may socially discredit a person [55], and with subsequent frameworks that conceptualize stigma as a process involving labeling, stereotyping, discrimination, and unequal power relations [56].

Physical manifestations were highly salient within the representational field. The frequent evocation of terms related to bodily damage suggests that participants perceived leprosy primarily through its visible consequences rather than through its biomedical characteristics or treatability.

Narratives from affected participants illustrate how these meanings operate in everyday life. One participant described concealing the hands and experiencing rejection after others noticed visible impairments:

- “*Sempre escondi as mãos*,* lembro que uma vez pediram ajuda*,* mas quando viram minhas mãos*,* recusaram ajuda.*”—translated: “*I always hid my hands*,* I remember once they asked for help*,* but when they saw my hands*,* they refused.*”

Another reported that people refused to accept money from them and that this discriminatory treatment led them to avoid eating in public places:

- “*Ninguém queria pegar meu dinheiro; nunca mais comi fora*,* deixei o dinheiro da xícara e do tamborete.*”—translated: “*Nobody wanted to take my money; I never ate out again*,* I left the money in the cup and on the stool.*”

Taken together, these accounts illustrate how visible bodily differences may lead to concealment, social avoidance, humiliation, and exclusion. Similar experiences were reported by other participants, who described hiding visible impairments or avoiding social interactions after experiencing or anticipating rejection. These patterns are consistent with studies demonstrating that visible impairments intensify enacted, anticipated, and internalized stigma [19, 57–59]. In many contexts, the social response to the disease is triggered less by knowledge about its transmission than by the visual recognition of bodily differences.

Individuals often adopt concealment strategies to protect their identity [60], while persistent impairments may sustain stigma even after treatment [51, 52, 61]. These dynamics indicate that stigma in leprosy is deeply embodied and cannot be addressed solely through biomedical interventions, requiring strategies that also target the social meanings attached to visible bodily differences, such as psychosocial support, community engagement, and public health actions [53].

### Delayed Care-Seeking and the Social Recognition of Disease

The prominence of bodily damage suggests that leprosy may become socially recognizable primarily through advanced and visible manifestations rather than through early symptoms or biomedical knowledge. The salience of “deformity” indicates that recognition may be anchored in visible impairment and historically constructed images of bodily transformation. This interpretation concerns the social recognition of disease and should not be conflated with the complete clinical diagnostic pathway.

The study directly assessed the interval between symptom recognition and care-seeking, rather than the interval from the first consultation to the confirmed diagnosis. Symptom interpretation, stigmatizing meanings, and structural barriers within health systems may shape delayed care-seeking [19, 62, 63]. When combined with health-system barriers, it may contribute to overall diagnostic delay and severe disability, including irreversible nerve damage and G2D [64, 65]. Participants’ accounts of prolonged intervals before seeking care illustrate how these processes may operate in practice.

Addressing delayed care-seeking and diagnostic delay therefore requires improving clinical awareness, supporting earlier disclosure and help-seeking, reshaping how early symptoms are socially interpreted, and strengthening primary healthcare systems through stigma-reduction and community-based education strategies.

### Knowledge Gaps and Institutional Vulnerabilities in Primary Healthcare

The study also identified important knowledge gaps regarding transmission, diagnosis, and treatment. Although 85.3% of affected participants recognized that leprosy is curable, 82.4% did not know how it is transmitted. Among the six who reported knowing, two clearly referred to close contact or respiratory transmission, one referred more broadly to an oral route, and the remaining responses cited heredity, shared surfaces, or hygiene.

Knowledge gaps were also evident among healthcare professionals. Three of 19 professionals (15.8%) reported not knowing how leprosy is transmitted, and some responses from those reporting knowledge were incomplete or inaccurate. Among the seven physicians, three (42.9%) reported not knowing the clinical forms of leprosy. Within this small professional sample, these findings indicate potential gaps in familiarity with early clinical presentation and may signal limitations in the diagnostic preparedness of primary healthcare services in an endemic setting. Similar findings have been reported elsewhere [14, 66].

Importantly, these institutional constraints may interact with the social dynamics identified in this study. When leprosy is socially associated with visible deformities and advanced disease, and professionals have limited experience diagnosing early cases, recognition may be delayed until later stages. This convergence reinforces patterns of delayed detection. Addressing this issue requires strengthening primary healthcare capacity through training, clearer guidelines, and better integration with specialized services [62, 67].

### Historical Memory, Institutional Segregation, and Structural Stigma

The historical context provides an important interpretive lens. Cruzeiro do Sul was historically associated with compulsory isolation, shaping collective experiences of the disease. SR are therefore influenced not only by contemporary experiences but also by historically sedimented narratives embedded in collective and institutional memory [21, 27]. In such contexts, a “person affected by leprosy” remains symbolically linked to exclusion and marginalization, even after policy changes [60, 68, 69]. These findings are consistent with broader historical analyses of leprosy control policies, which demonstrate how institutional practices have contributed to the long-term reproduction of stigma within health systems [68, 69].

This persistence is consistent with structural stigma, in which discriminatory meanings are not confined to interpersonal attitudes but are embedded in institutional arrangements, professional routines, and patterns of care [53]. In the case of leprosy, structural stigma may manifest through limited professional training, weak contact surveillance, or the continued circulation of stigmatizing representations within healthcare systems. Evidence from multiple settings shows that discriminatory experiences may occur even within healthcare settings, reinforcing mistrust and delaying care-seeking [19, 54, 70]. Thus, stigma should be understood not only as interpersonal but also as structurally produced.

### Contributions to Public Health

The findings reinforce the need to understand leprosy as a biosocial phenomenon shaped by structural inequality, institutional practices, and socially constructed meanings. Effective responses therefore require integrated strategies that address both biomedical control and the social production of stigma. Despite progress, Brazil remains among the countries with the highest leprosy burden, highlighting ongoing challenges in achieving the WHO 2030 targets, including a 70% reduction in annual new cases and a 90% reduction in the rate of new cases with G2D [71].

Interventions should promote early symptom recognition through culturally sensitive educational initiatives while addressing the symbolic dimensions of the disease. Efforts to reduce stigma must extend beyond correcting misconceptions to include psychosocial support and community-based strategies that challenge historically rooted narratives of deformity, danger, and exclusion [19]. Strengthening primary healthcare remains essential, particularly through continuous professional training, improved diagnostic capacity, and more effective contact surveillance [66, 72].

In historically endemic settings, addressing the legacy of segregation and fostering community engagement may contribute to transforming persistent stigmatizing narratives. Beyond leprosy, these findings highlight how stigma becomes embodied, how diagnostic delay is socially structured, and how institutional arrangements and historical memory shape health system responses.

### Strengths and Limitations

A major strength of this study is the inclusion of perspectives from both persons affected by leprosy and primary healthcare professionals, allowing comparison between experiential and service-based viewpoints. The structural analysis of social representations provided a systematic account of shared meanings related to stigma and disease visibility. Several limitations should be considered. The study was conducted in a single municipality, which limits its transferability to other settings. Sampling procedures differed between groups and were designed to capture relevant perspectives rather than to produce population-representative estimates; therefore, selection bias cannot be excluded. Some variables had missing or noninterpretable responses, and descriptive statistics were calculated using available-case denominators. The small professional sample, including only seven physicians, limits inferences about primary healthcare preparedness. Prototypical analysis identifies candidate structural elements but does not establish centrality without complementary procedures. Finally, the cross-sectional design captures data at a single point in time and cannot determine temporal change or causal relationships [73].

## Conclusion

Leprosy remains socially anchored in stigma, embodied visibility, and exclusion, shaping how the disease is recognized and potentially contributing to delayed diagnosis despite its biomedical curability. These findings highlight that stigma cannot be reduced to knowledge gaps alone, but reflects the interplay of socially constructed meanings, embodied experience, and institutional dynamics. Addressing leprosy, therefore, requires more than interrupting transmission; it demands integrated strategies that strengthen primary healthcare while transforming the social and institutional conditions that sustain stigma, diagnostic delay, and exclusion.

## Supplementary Information

Below is the link to the electronic supplementary material.

Supplementary Material 1. Online Resource 1. Scientific Research Instrument for Persons Affected by Leprosy. Structured interview and Free Word Association Test used to assess sociodemographic characteristics, experiences, knowledge, care-seeking, stigma, and social representations of leprosy among participants previously diagnosed with the disease

Supplementary Material 2. Online Resource 2. Scientific Research Instrument for Healthcare Professionals. Structured interview and Free Word Association Test used to assess professional characteristics, training, knowledge, practices, diagnostic experience, and social representations of leprosy among primary healthcare professionals

## Data Availability

The data supporting the findings of this study are not publicly available because they contain sensitive information and their disclosure could pose a risk of participant re-identification. De-identified data may be made available by the corresponding author (F.A.A.) upon reasonable request and subject to compliance with Brazilian Resolution *CNS* No. 466, dated December 12, 2012, and other applicable ethical requirements.
