# Supplementary material for: Leprosy-Related Stigma and Social Representations Among Persons Affected by Leprosy and Primary Healthcare Professionals: A Structural Analysis in the Brazilian Amazon
Source: J Community Health. 2026 Jul 25;51(6):976–90. doi: 10.1007/s10900-026-01618-2 (PMC13748295; doi:10.1007/s10900-026-01618-2)
Supplement: Supplementary file 1 [file 10900_2026_1618_MOESM1_ESM.pdf]

Supplementary Material 1. Scientific Research Instrument – Patients

SCRIPT FOR FREE WORD ASSOCIATION STIMULUS

Block A - INDUCING STIMULUS

a1. What words come to mind when I say "leprosy"? Tell me 5 words (put them in the order she says them)

1st ______________________________

2nd ______________________________

3rd ______________________________

4th ______________________________

5th ______________________________

a2. Of the words you said to me, put them in order from the most important to the least important to you (write in the order she says)

1st ______________________________

2nd ______________________________

3rd ______________________________

4th ______________________________

5th ______________________________

a3. What image comes to mind when I say the word "leprosy"?

____

____

____

____

____

____

____

____

Block C - SOCIOECONOMIC PROFILE OF THE PATIENT

c1. Sex: O female O male

c2. Age: ___________________________

c3. Skin color/ethnicity: O black O mixed O white O yellow O indigenous

c4. Level of education:

O never studied O literate O former primary

O elementary school (or 1st grade incomplete) O elementary school (or 1st grade complete)

O high school (or incomplete secondary school) O high school (or completed secondary school)

O incomplete higher education O complete higher education

c5. Marital status: O married O single O consensual union

O widowed O divorced O legally separated

c6. Who do you live with? O husband/wife or partner O children

O others ____

c7. Do you work outside the home? O yes O no

c8. What do you do? ____

FINANCIAL CONDITION:

c9. Do you live independently, without relying on anyone? O yes O no

c9.1. If no: O partially dependent O totally dependent

c10. Who helps you financially?

O husband/wife or partner O your father or mother O relatives O friends

O others: ____

Block D - GUIDING QUESTIONS

d1. Do you know how leprosy is transmitted? ○ yes ○ no

d2. If yes, how do you think leprosy is transmitted?

____

____

____

____

____

d3. How did you find out about leprosy?

○ in a health center ○ TV campaign ○ through neighbors

○ spouse/partner ○ someone in your family ○ at work ○ never heard of it

○ others: ____

d4. Do you think leprosy can be cured? ○ yes ○ no

d5. How long ago were you diagnosed with leprosy? ______________________________

d6. How long did it take you to seek help? ______________________________

d7. What did it mean for you to be diagnosed with leprosy? What was the impact?

____

____

____

____

____

____

____

____

____

d8. Have there been any changes in your work and social interactions with your work colleagues? Which ones?

d9 Have there been any changes in your family? What about your partner? Which ones?

d10. Has anything changed regarding your friends? Which ones?

d11. Have there been any changes in your neighborhood? Which ones?

____

____

____

____

____

____

____

d12. What was leprosy, and what is it today in your view?

____

____

____

____

____

d13. How do you see the care provided by healthcare professionals?

____

____

____

____

____

____

d14. Is there supervision and assistance for the treatment and control of communicants?

○ always ○ often ○ occasionally ○ rarely ○ never

Date on which the interview took place:

____/____/2022
