# Supplementary material for: Leprosy-Related Stigma and Social Representations Among Persons Affected by Leprosy and Primary Healthcare Professionals: A Structural Analysis in the Brazilian Amazon
Source: J Community Health. 2026 Jul 25;51(6):976–90. doi: 10.1007/s10900-026-01618-2 (PMC13748295; doi:10.1007/s10900-026-01618-2)
Supplement: Supplementary file 2 [file 10900_2026_1618_MOESM2_ESM.pdf]

Supplementary Material 2. Scientific Research Instrument – Healthcare Professionals

SCRIPT FOR STIMULUS INDUCING FREE ASSOCIATION OF WORDS

Block A - INDUCING STIMULUS

a1. What words come to mind when I say "leprosy"? Tell me 5 words (put them in the order she says them)

1st ____

2nd ____

3rd ____

4th ____

5th ____

a2. Of the words you said to me, put them in order from the most important to the least important to you (write in the order she says)

1st ____

2nd ____

3rd ____

4th ____

5th ____

Block B - MENTAL IMAGE INDUCING STIMULUS

b1. What image comes to mind when you say "leprosy"? (justify)

Block C - SOCIOECONOMIC PROFILE OF THE HEALTHCARE PROFESSIONAL

c1. Sex: ○ female ○ male

c2. Age: ____________________

c3. Level of education:

○ never studied ○ literate ○ former primary

○ elementary school (or 1st grade incomplete) ○ elementary school (or 1st grade complete)

○ high school (or incomplete secondary school) ○ high school (or completed secondary school)

○ incomplete higher education ○ complete higher education

c4. How long have you been working as a healthcare professional? ____________________

Block D - GUIDING QUESTIONS

d1. Have you attended any courses or training on health actions in primary care regarding leprosy?

○ yes ○ no

d1.1. If yes, how long was the course or training, in hours? ____________________

d2. Do you think leprosy can be cured? ○ yes ○ no

d3. Do you know how leprosy is transmitted? ○ yes ○ no

d3.1. If yes, how do you think leprosy is transmitted?

____

____

____

____

____

____

d4. Have you ever had the opportunity to diagnose/report an individual with suspected leprosy?

○ yes ○ no

d5. Can you describe the main signs and symptoms of leprosy?

○ yes ○ no

d5.1. Which ones?

____

____

____

____

____

____

d6. What actions do you take in the Healthcare Unit or in the community (during home visits, etc.) to contribute to the control and treatment of leprosy?

____

____

____

____

____

____

____

____

d7. Is there supervision and assistance for the treatment and control of communicants?

○ always ○ often ○ occasionally ○ rarely ○ never

d8. Do you know the clinical forms of leprosy? ○ yes ○ no

d8.1. Which ones?

____

____

____

d9. In your opinion, which of these drugs are recommended for the paucibacillary treatment of leprosy?

- ○ Rifampicin and Dapsone
- ○ Clofazimine only
- ○ Rifampicin and Clofazimine
- ○ Rifampicin only
- ○ Rifampicin, Clofazimine, and Dapsone

d10. In your opinion, which of these drugs are recommended for the multibacillary treatment of leprosy?

- ○ Rifampicin only
- ○ Clofazimine only
- ○ Rifampicin, Dapsone, and Clofazimine
- ○ Dapsone only

Date on which the interview took place:

____/____/2022
